# Knowledge and awareness of cervical cancer and human papillomavirus vaccination among female university students

**DOI:** 10.4102/safp.v66i1.5885

**Published:** 2024-07-16

**Authors:** Charles C. Ndubuisi, Olga Maphasha, Sunday O. Okeke

**Affiliations:** 1 Department of Family Medicine, Faculty of Health Sciences, University of Pretoria, Pretoria, South Africa; 2 Department of Family Medicine & Primary Health Care, Faculty of Medicine, Sefako Makgatho Health Science University, Pretoria, South Africa

**Keywords:** cervical cancer, human papillomavirus, Pap smear, human papillomavirus vaccines, knowledge

## Abstract

**Background:**

Prevention strategies for reducing cervical cancer incidence rely on informed populations, particularly those most at risk. This study assesses the knowledge and awareness of female university students towards cervical cancer, human papillomavirus (HPV) and its vaccination.

**Methods:**

A validated self-administered questionnaire was used in a descriptive cross-sectional study among female university students. The data were analysed with Statistical Package for Social Sciences version 26, and *p* < 0.05 was considered significant.

**Results:**

The total participants were 190 with a mean age of 22.6 ± 4.35 years. The majority (90%) were aware of cervical cancer, and 78.9% agreed it is a terminal illness, but fewer participants knew it was associated with infection (63.7%), and that it had effective risk-reducing methods (70.5%). Only 32.6% were aware of the Pap smear test, less than half (43.2%) were aware of the cervical cancer vaccine and only 43.7% knew it was available locally. Although fewer (39.5%) considered themselves susceptible to cervical cancer, many (62.1%) would like a Pap smear test. Overall, 88.9% of the participants possessed adequate knowledge of cervical cancer, 67.9% of the HPV vaccine and only 33.7% of HPV. Ethnicity (*p* = 0.03), year of study (*p* = 0.001) and institution (*p* = 0.002) were all significantly associated with knowledge levels, vaccine awareness and Pap smear test awareness.

**Conclusion:**

Participants showed low HPV knowledge and varying awareness levels regarding cervical cancer, HPV and HPV vaccine.

**Contribution:**

This study provides insights into female university students’ knowledge and awareness gaps, highlighting the need for targeted interventions.

## Introduction

Cervical cancer is a global health problem being the fourth most frequent cancer seen in females.^1,2^ About 600 000 cases are seen on an annual basis worldwide, and recent trends suggest that by the year 2030, more than 700 000 new cases of cervical cancer will be recorded globally, and most of these are likely to be seen in developing nations.^1,2^ Within the Sub-Saharan African region, cervical cancer remains a leading cause of morbidity and mortality among women.^1,2,3^ The persistent infection with the human papillomavirus (HPV), a double-stranded deoxyribonucleic acid (DNA) virus that primarily spreads through skin-to-skin and sexual contact in which it mainly targets the epithelial cells including those in the cervix,^4^ has been shown to be the major risk factor.^1,2,3^ This is evident in the age-standardised incidence of 75 per 100 000 in high-risk nations compared to 10 per 100 000 in the developed world, which are often the low-risk countries.^1,3^ This also explains why 90% of cervical cancer-related deaths worldwide are reported by low-income countries.^1,2,3^ Cervical cancer primarily affects women in their fifth or sixth decade, around age 54 years, with a precancerous stage occurring in those under 40 years.^1,2^ Some of the independent risk factors include early sexual activity, multiple male partners, male partners with multiple partners, early childbirth, multiple pregnancies, smoking, prolonged oral contraceptive use and weakened immune systems.^1^ Although an infection with HPV is a necessary but not sufficient cause of cervical cancer, nearly all cases of the malignancy can be attributable to HPV-linked infection, particularly the HPV strains 16 and 18, which are known to be associated with nearly 70% of all invasive cervical cancer cases globally.^1,3^

There is a general consensus that countries in sub-Saharan Africa have a pressing need for broader and better education on the issue of HPV infection, as well as cervical cancer screening and prevention.^5,6^ This has necessitated the World Health Organization (WHO) to declare a global strategy to eliminate cervical cancer as a public health problem by the year 2030.^1,2,3^ Already, vaccines against HPV 16 and 18 have been approved for use in young people worldwide. The overarching aim of this vaccination is to achieve primary prevention of HPV infection. Moreover, the expansion of this programme is laden with an intent to reduce transmission of HPV through preventive vaccination for females before sexual debut and to screen for precancerous lesions among sexually active females who are at risk of developing cervical cancer and referral to sources of further diagnostic care and treatment when abnormal lesions are found.^5,6^ According to the 2021 report from the Institute of Oncology (ICO) or the International Agency for Research on Cancer (IARC), the risk of cervical cancer was very high among a population of nearly 21.3 million females in South Africa who are 15 years and older.^3^ In particular, women aged between 15 years and 44 years have been shown to have the highest burden of the malignancy. This risk is partly because of the fact that at every given time 3.2% of women in their reproductive age carry the cervical HPV–16 or - 18 infection, which is evident in the high proportion (64.2%) of invasive cervical cancers that have been linked to HPVs strains 16 or 18.^2,3^ Therefore, it is not surprising that there are 10 702 new cases of cervical cancers diagnosed annually in South Africa, out of which about 5870 women are likely to die from this disease.^2,6^

Despite government policy changes, screening uptake remains low, and there is a high loss to follow-up for women with abnormal Pap smear tests.^4,5,6,7,8^ The young female students attending tertiary institutions in Pretoria are among the tens of millions of women who fall within the age category that is considered to be at high risk of cervical cancer in South Africa.^2,3^ Without a doubt, we have a huge burden of cervical cancer in South Africa, which underscores the rationale for embarking on research into the disease and its associated risk factors at this point. Moreover, the unacceptably high morbidity and mortality rates associated with cervical cancer imply that the problem probably exists in terms of levels of awareness, accessibility, availability or acceptability of the current cervical cancer prevention services by the female population.^4^ Notwithstanding the level of cervical cancer and HPV vaccination knowledge possessed by these university female students, knowledge alone is not sufficient for one to act. Knowledge and awareness are constructs of health determinants known to influence the health behaviour of most at-risk people with regards to the acceptability of healthcare services such as vaccination, screening, et cetera. As stated above, HPV is a common sexually transmitted infection known to be the leading cause of cervical cancer; therefore, knowledge and awareness of HPV, its transmission and associated risks are crucial for the prevention of cervical cancer. It is upon this premise that the current study was conceived with the aim of determining the level of knowledge and awareness of female university students in Pretoria regarding cervical cancer, HPV and its vaccination.

## Research methods and design

### Design and setting

The research design used was a cross-sectional descriptive-analytical study, which was conducted between June 2023 and September 2023 among female students attending various universities in Pretoria. The study setting chosen was Pretoria, South Africa, the capital city with a large metropolitan area within the City of Tshwane municipality, it has a vibrant youth population and is one of the heterogeneous geographical areas in South Africa where all the racial ethnicities could be found.^7^ Young people account for a large proportion of the people living in Pretoria, with about 40% of the total population classified as youth, making it one of the ‘youngest’ cities in the country.^7^

### Target population

For this study, the target population was female students attending a university in Pretoria, and three out of the four universities in Pretoria were chosen. These were the University of Pretoria, Sefako Makgatho Health Sciences University and Tshwane University of Technology. The University of South Africa (UNISA) was not considered because it is mainly a distance learning institution, which might prove challenging during data collection. Nevertheless, the three institutions chosen for this research were deemed appropriate given that their student populations represent a good spread of the various racial groups found in South Africa.^9^

### Sample and sampling

The Raosoft online sample size calculator was used to calculate the sample size that represents the study population, the Raosoft online sample size calculator was used.^10^ A minimum sample size of 162 participants was needed based on the Raosoft online sample size calculator (0.05 margin of error at 95% confidence interval (CI) and 88% response distribution) and an estimated population of 15 000 female university students in the three universities.^9^ An adjustment was made for non-response or incomplete questionnaires using an anticipated response rate of 85%, (*N*_1_ = *N*_0_/0.85 = 190). Proportional quota sampling was used to recruit consenting participants, which ensured the normal spread of participants across the three institutions and minimised clustering. Recruitment was conducted by posting recruitment messages on WhatsApp groups and sending emails to class groups and lecturers. The researcher gave all female participants equal opportunity to participate in the study.

### Data collection

The research instrument used was the Cervical Cancer Knowledge Prevention–64 (CCKP–64) questionnaire,^11^ a validated tool originally designed to measure knowledge of cervical cancer and its associated factors among young women,^11,12^ CCKP–64 is modifiable and has been used in previous studies.^13,14,15,16,17,18,19^ Only participants who provided informed consent received hard copies of the questionnaires. Furthermore, a soft copy in the form of an electronic version of the research questionnaire was made available online through a weblink for those who preferred to complete an electronic copy of the questionnaire. Questionnaires were distributed to participants, and all were administered in English, the language of teaching and training at these universities. Participants’ information leaflets were given to each participant beforehand, and this served to inform them about the study and what were the requirements in terms of their participation. However, those who elected to complete the electronic version were able to read the online information leaflet. A total of 196 completed questionnaires were received, 141 hardcopies questionnaires and 55 from online submissions. Six questionnaires that only had demographic information completed were found unsuitable for analysis from the hardcopies and were excluded from capturing which resulted in 190 questionaries being used eventually.

### Data analysis

The data were captured onto an Excel spreadsheet after the questionnaires were thoroughly checked for errors through manual verification of completed questionnaires and sorting by the biostatistician. The data capturing onto the spreadsheet was done using a double-entry method to minimise errors and enhance data accuracy. Subsequently, the data were imported into Statistical Package for Social Sciences (SPSS) statistical version 25 for analysis. Statistical Package for Social Science’s frequency function was employed to assess individual variables for data accuracy, identifying missing or out-of-range values, as well as univariate outliers. Normality tests revealed a non-normal distribution of data, precluding the use of parametric tests for associations. Descriptive statistics were done and presented as means, proportions, frequency tables and percentages. A test of significance was performed on the data, where appropriate, and any observed differences were considered statistically significant at a *p*-value of less than 0.05.

### Ethical considerations

Ethical clearance to conduct this study was obtained from the University of Pretoria, Faculty of Health Sciences Research and Ethics Committee (FHREC) (reference no. 26/2023). In addition, the participants were required to provide written informed consent before they participated in the study.

## Results

A total of 190 female university students made up the participants in this study; their sociodemographic characteristics are summarised in Table 1. The mean age of participants was 22.6 years (standard deviation [s.d.]: = 4.35), and the median was 21.5 (range: 18–48) years. Most participants were South African nationals (92.1%) and were above 21 years old (74.2%). Almost all the participants were black people (80.0%), studying full-time at their institutions (93.2%). More than half (56.3%) of the participants relied on the Internet for information on cervical cancer, followed by 38 (18.9%) who relied on medical doctors.

**TABLE 1:** Sociodemographic characteristics of the participants (*N* = 190).

Variable	Frequency (*n*)	%	Mean	s.d.
**Age (years)**	-	-	22.6	4.35
**Age group (years)**				
< 21	49	25.8	-	-
≥ 21	141	74.2	-	-
**Tertiary institution**				
University of Pretoria	95	50.0	-	-
Sefako Makgatho Health Sciences University	26	13.7	-	-
Tshwane University of Technology	69	36.3	-	-
**Ethnicity**				
Black people	152	80.0	-	-
White people	31	16.3	-	-
Mixed race people	5	2.6	-	-
Indian/Asian people	2	1.1	-	-
**Study type**			-	-
Full-time	177	93.2	-	-
Part-time	13	6.8	-	-
**Course of study**				
Degree	135	71.1	-	-
Diploma	49	25.8	-	-
Certificate	4	2.1	-	-
Others	2	1.1	-	-
**Year of study**				
Year 1	30	15.8	-	-
Year 2	35	18.4	-	-
Year 3	78	41.1	-	-
Year 4	39	20.5	-	-
Year 5	3	1.6	-	-
Postgraduate	5	2.6	-	-
**Nationality**				
South African	175	92.1	-	-
Non-South African	15	7.9	-	-
**Source of cervical cancer information**				
Internet	107	56.3	-	-
Television	6	3.2	-	-
Newspaper	1	0.5	-	-
Doctors	36	18.9	-	-
Leaflets	3	1.6	-	-
School	21	11.1	-	-
Family	8	4.2	-	-
Others	8	4.2	-	-

s.d., standard deviation.

Table 2 represents the participants’ general knowledge about questions related to the primary and secondary prevention of cervical cancer, most participants were aware of cervical cancer (90%) and agreed that it can be a terminal illness (78.9%), but a few were not sure about its association with an infection (27.9%). The majority (70.5%) acknowledged the existence of effective cervical cancer risk reduction methods. Only a small proportion of the participants (8.4%) have encountered cervical cancer among their relatives or friends; however, a sizable proportion (40%) believed that cervical cancer could affect them in future.

**TABLE 2:** General knowledge about cervical cancer (*N* = 190).

Questions	Yes		No		Don’t know	
	*n*	%	*n*	%	*n*	%
Have you ever heard of cervical cancer?	171	90.0	15	7.9	4	2.1
Can cervical cancer be a terminal illness	150	78.9	8	4.2	32	16.9
Can cervical cancer be associated with an infection?	121	63.7	16	8.4	53	27.9
Is there an effective method that significantly reduces its risk?	134	70.5	6	3.2	50	26.3
Have you ever had direct contact with the disease	16	8.4	160	84.2	14	7.4
Do you think this disease could affect you in the future?	75	39.5	38	20.0	77	40.5
Have you heard about the vaccine ‘against cervical cancer’?	82	43.2	107	56.3	1	0.5
If such a vaccine exists, is it available in South Africa?	83	43.7	19	10	88	46.3
Is it free of charge?	43	22.6	40	21.1	107	56.3
Does it guarantee 100% protection from cervical cancer?	12	6.3	85	44.7	93	48.9
Do you know where you can get vaccinated?	60	31.6	67	35.3	63	33.2
Have you ever been vaccinated?	37	19.5	136	71.6	17	8.9
Have you ever heard about Pap smear test?	62	32.6	128	67.4	-	-
Is it a test that gives a 100% chance of early diagnosis of cervical cancer?	42	22.1	29	15.3	119	62.6
Is the test painful?	19	10	47	24.7	124	65.3
Is it a time-consuming test?	14	7.4	71	37.4	105	55.3
Is it possible to be tested free of charge?	70	36.8	15	7.9	105	55.3
Is it sufficient to do the test only once in order to eliminate the risk of cervical cancer?	25	13.2	82	43.2	83	43.7
Can the test cause serious complications?	11	5.8	69	36.3	110	57.9
Is it possible for Pap smear to increase the susceptibility to cervical cancer in the future?	34	17.9	79	41.6	77	40.5
Do you think you should undergo Pap smear test	118	62.1	20	10.5	52	27.3

*Source:* Adapted from Jaglarz K, Tomaszewski KA, Kamzol W, Puskulluoglu M, Krzemieniecki K. Creating and field-testing the questionnaire for the assessment of knowledge about cervical cancer and its prevention among schoolgirls and female students. J Gynecol Oncol. 2014;25:81–89. https://doi.org/10.3802/jgo.2014.25.2.81

Less than half (43.2%) of the participants have heard about the cervical cancer vaccine, and almost the same number perceived it to be available locally (43.7%) or believed in its 100% efficacy (44.7%). In addition, very few (22.6%) participants agreed that the vaccine was available free of charge or knew where to receive it (31.6%) and fewer (19.5%) of the participants indicated that they have received the vaccine. Regarding Pap smear tests, only a few participants (32.6%) have heard about them although most participants (62.1%) would like to undergo the Pap smear test. In addition, only a few of the participants knew that it was possible to obtain a Pap smear test for free (36.8%) in South Africa.

The risk factors associated with cervical cancer were assessed with a Likert scale ranging from a weak relationship to a strong relationship on a scale 0–5. This is shown in Table 3, which shows participants’ responses in two categories based on the perceived strength of the relationship between each itemised risk factor and cervical cancer. Factors such as genetic factors (74.7%), HPV infection (77.9%) and multiple sexual partners (76.3%) were correctly recognised by a substantial proportion as having a strong relationship with cervical cancer.

**TABLE 3:** Knowledge of risk factors associated with cervical cancer (*N* = 190).

Risk factors	Weak relationship		Strong relationship	
	*n*	%	*n*	%
1 Young age	128	67.4	62	32.6
2 Genetic factors	48	25.3	142	74.7
3 Human papillomavirus infection	42	22.1	148	77.9
4 Human immunodeficiency virus infection	64	33.7	126	66.3
5 Multiple sexual partners	45	23.7	145	76.3
6 Early sexual initiation	79	41.6	111	58.4
7 History of sexually transmitted diseases	54	28.4	136	71.6
8 Alcohol abuse	134	70.5	56	29.5
9 Smoking	120	63.2	70	36.8
10 Miscarriages and abortions	66	34.7	124	65.3
11 Multiparty	102	53.7	88	46.3
12 Early menarche	136	71.6	54	28.4
13 Use of condoms	164	86.3	26	13.7
14 Hormonal contraception	117	61.6	73	38.4
15 Breast feeding	171	90	19	10
16 Use of drugs or psychoactive substances	114	60	76	40
17 Use of public swimming pools	132	69.5	58	30.5

Table 4 shows the participants’ general knowledge of HPV infection and HPV vaccination. Most of the participants correctly identified HPV (93.2%) and multiple sexual partners (89.5%) as possible causes of cervical cancer, and that HPV-positive tests will not always result in cervical cancer (84%). Additionally, when asked if ‘someone who has had HPV vaccine cannot develop cervical cancer’, nearly all the participants (86.8%) correctly indicated the statement as false. On the other hand, very few participants 32 (16.8%) know that HPV does not always need treatment and that HPV testing is not used to determine whether the HPV vaccine is needed (40%). About half of the participants (52%) know that an HPV test cannot tell how long one has been infected with HPV.

**TABLE 4:** Knowledge of human papillomavirus and human papillomavirus vaccine (*N* = 190).

Knowledge of human papillomavirus (HPV)	Correct answer	
	*n*	%
1 HPV is very rare **(F)**	134	70.5
2 HPV always has visible signs or symptoms **(F)**	107	56.3
3 HPV can cause cervical cancer **(T)**	177	93.2
4 HPV can be passed on by genital skin-to-skin contact **(T)**	132	69.5
5 There are many types of HPV **(T)**	160	84.2
6 HPV can cause HIV or AIDS **(F)**	133	70.0
7 HPV can be passed on during sexual intercourse **(T)**	169	88.9
8 HPV can cause genital warts **(T)**	160	84.2
9 Men cannot get HPV **(F)**	149	78.4
10 Using condoms reduces the risk of getting HPV **(T)**	165	86.8
11 HPV can be cured with antibiotics **(F)**	107	56.3
12 Having many sexual partners increases the risk of getting HPV **(T)**	170	89.5
13 HPV usually does not need any treatment **(T)**	32	16.8
14 Most sexually active people will get HPV at some point in their lives **(T)**	94	49.5
15 A person could have HPV for many years without knowing it **(T)**	156	82.1
16 Having sex at an early age increases the risk of getting HPV **(T)**	130	68.4
17 An HPV test can tell how long you have had an HPV infection **(F)**	99	52.1
18 If a woman tests positive for HPV, she will definitely get cervical cancer **(F)**	163	85.8
19 An HPV test can be done at the same time as a Pap Smear test **(T)**	160	84.2
20 HPV testing is used to indicate whether the HPV vaccine is needed **(F)**	76	40.0
21 When you have an HPV test, you get the results the same day **(F)**	103	54.2
22 If HPV test shows that a woman does not have HPV, her risk of cervical cancer is low **(T)**	96	50.5
23 HPV vaccines require three doses **(T)**	126	66.3
24 The HPV vaccines offer protection against all sexually transmitted infections **(F)**	135	71.1
25 The HPV vaccines are most effective if given to people who have never had sex **(T)**	86	45.3
26 Someone who has had HPV vaccine cannot develop cervical cancer **(F)**	165	86.8
27 The HPV vaccines offer protection against most cervical cancers **(T)**	129	67.9
28 One of the HPV vaccines offers protection against genital warts **(T)**	140	73.7
29 Girls who got the HPV vaccine do not need Pap smear test when they are older **(F)**	165	86.8

HPV, human papillomavirus; F, false; T, true.

Table 5 shows the aggregate knowledge scores on a percentage point for each participant; this was calculated based on the number of correctly answered questions regarding cervical cancer-related questions, HPV-related questions and HPV vaccine-related questions. Participants who correctly answered 70% of the items on cervical cancer-related questions and HPV vaccine-related questions were considered to have adequate knowledge in that respective domain. Meanwhile, adequate knowledge of HPV-related questions required that the participants correctly answer 80% of the questions. As shown in Table 5, most of the participants demonstrated adequate knowledge of cervical cancer (89%) and the HPV vaccine (67.9%), but only a few (33.7%) of the female students possessed adequate knowledge regarding HPV.

**TABLE 5:** Summary of participants’ knowledge scores (*N* = 190).

Knowledge domain	Frequency	%
**Cervical cancer knowledge score**		
< 70%	21	11.1
≥ 70%	169	88.9
**HPV knowledge score**		
< 80%	126	66.3
≥ 80%	64	33.7
**HPV vaccine knowledge score**		
< 70%	61	32.1
≥ 70%	129	67.9

HPV, human papillomavirus.

Table 6 contains the results of Chi-squared tests done to determine factors associated with participants’ knowledge scores along the three domains of cervical cancer-related knowledge, HPV-related knowledge and HPV vaccine-related knowledge. Type of study (*p* = 0.04), year of study (*p* = 0.01), tertiary institution (*p* = 0.02) and ethnicity (*p* = 0.03) were significantly associated with participants’ cervical cancer-related knowledge. On the HPV knowledge domain, course of study (*p* = 0.026) and tertiary institution (*p* < 0.0001) were strongly associated with HPV knowledge score. Tertiary institution (*p* = 0.002), ethnicity (*p* = 0.001) and course of study (*p* = 0.02) were the only independent variables shown to be associated with participants’ knowledge of the HPV vaccine.

**TABLE 6:** Factors associated with participants’ knowledge scores (*N* = 190).

Factors	Cervical cancer knowledge score					HPV knowledge score					HPV vaccine knowledge score				
	< 70%		≥ 70%		*p*	< 80%		≥ 80%		*p*	< 70%		≥ 70%		*p*
	*n*	%	*n*	%		*n*	%	*n*	%		*n*	%	*n*	%	
**Age group (years)**															
< 21	45	91.8	38	77.6	0.60	18	36.7	31	63.3	0.053	11	22.4	4	8.2	0.478
≥ 21	124	87.9	88	62.4	-	43	30.5	98	69.5	-	53	37.6	17	12.1	-
**Tertiary institution**															
UP	83	87	67	70.5	0.02	26	27.4	69	72.6	0.0001	28	29.5	12	12.6	0.002
SMU	20	76.9	7	26.9	-	3	11.5	23	88.5	-	19	73.1	6	23.1	-
TUT	66	95.7	52	75.4	-	32	46.4	37	53.6	-	17	24.6	3	4.3	-
**Ethnicity**															
Black people	139	91.4	105	69.1	0.03	57	37.5	95	62.5	0.035	47	30.9	13	8.6	0.001
Non-Black people	30	78.9	21	55.3	-	4	10.5	34	89.5	-	17	44.7	8	21.1	-
**Study type**															
Full-time study	160	90.4	116	66.5	0.041	57	32.2	120	67.8	0.548	61	34.5	17	9.6	1.00
Part-time study	9	69.2	10	76.9	-	4	30.8	9	69.2	-	3	23.1	4	30.8	-
**Course of study**															
Degree courses	117	86.7	83	61.5	0.078	36	26.7	99	73.3	0.026	52	38.5	18	13.3	0.02
Non-degree courses	52	94.5	43	78.2	-	25	45.5	30	54.5	-	12	21.8	3	5.5	-
**Year of study**															
Year 1 to 3	132	92.3	107	74.8	0.010	51	35.7	92	64.3	0.0001	36	25.2	11	7.7	0.067
≥ Year 4	37	78.7	19	40.4	-	10	21.3	37	78.7	-	28	59.6	10	21.3	-
**Nationality**															
RSA citizen	157	89.7	117	66.9	0.221	55	31.4	120	68.6	0.590	58	33.1	18	10.3	0.567
Non-RSA citizen	12	80.0	9	60.0	-	6	40.0	9	60.0	-	6	40.0	3	20.0	-

UP, University of Pretoria; SMU, Sefako Makgatho Health Sciences University; TUT, Tshwane University of Technology; RSA, Republic of South Africa.

Furthermore, on bivariate analyses, HPV vaccine awareness was found to be associated with tertiary institution (*p* = 0.03), race (*p* < 0.0001), year of study (*p* = 0.002) and course of study (*p* = 0.04). Likewise, tertiary institution (*p* < 0.0001), ethnicity (*p* = 0.03), course of study (*p* = 0.001) and year of study (*p* = 0.001) showed statistically significant association with Pap smear test awareness among the participants.

## Discussion

Overall, the study findings indicate a relatively high level of cervical cancer knowledge and awareness among the participants, as the majority (90.0%) have heard of cervical cancer, indicating a very high level of awareness; moreover, participants also exhibited varying levels of knowledge concerning risk factors associated with cervical cancer; in particular, there is evidence of poor knowledge regarding HPV and HPV vaccine. This poor knowledge of HPV is concerning; even though almost all the participants were aware of cervical cancer, little is known about Pap smear tests and that the test was available free of charge. Any uncertainty regarding the cost of the vaccine could impact individuals’ decision-making when it comes to seeking vaccination, emphasising the importance of providing clear information about vaccine accessibility and affordability.^15,18,20^ The Pap smear test is arguably regarded as the most effective tool in secondary prevention of cervical cancer in a form of screening, yet nearly 70% of the participants claimed they had not heard about this screening test, and only 62% think they needed to undergo a Pap smear test. This observation closely aligns with findings from diverse studies conducted across distinct settings, revealing a pervasive lack of knowledge regarding cervical cancer, HPV and HPV vaccine awareness.^16,17,18,19,21^ These findings have implications for future activities in relation to the prevention of cervical cancer in the setting of this study.

Another notable finding was that only one-third (33.7%) of the participants knew enough about HPV, whereas HPV is considered as the most important risk factor for cervical cancer.^1,4^ These findings support what previous studies have revealed, especially those conducted among undergraduate students and young women.^7,15,16,17,18,19^ For example, a study among undergraduate students in Ghana showed poor knowledge among the participants regarding the link between HPV and cervical cancer.^15^ Another study among female university students in Nigeria revealed that only 30% and 13.5% of the participants were aware of HPV and the HPV vaccine, respectively.^17^ Other studies have also shown similar trends, where limited knowledge of HPV and the HPV vaccine and other cervical cancer prevention services were reported by the participants.^19,21,22,23,24^ Similar to what the studies mentioned here have shown, a study conducted in South Africa among nurses also revealed little knowledge concerning the role of HPV infection and the HPV vaccine, despite the nurses exhibiting a commendable awareness of cervical cancer.^7^ Research indicates that the discernible disparities in comprehension among these young female participants often stem from deficient general knowledge, prevalent misconceptions and an absence of public health campaigns and educational programmes.^20^

Given that some previous studies have reported varying levels of awareness and knowledge regarding cervical cancer and its risk factors,^21,22,23,24^ in the current study, there were some misconceptions and a gap in the knowledge regarding some important risk factors associated with cervical cancer, which appeared to be unknown to many of the participants. Notably, a considerable proportion of participants held misconceptions regarding HPV vaccines, such as believing they offer protection against all sexually transmitted infections and ensure immunity against cervical cancer. Additionally, some did not know that HPV vaccines are most effective when administered to individuals who have never had sex, and that they offer protection against most cervical cancers. Besides, a significant number of participants erroneously believed that girls who received the HPV vaccine do not require Pap smear tests when they are older. However, it is encouraging to note that a significant proportion of participants recognised the role of sexual behaviour in HPV transmission. For instance, a high percentage correctly identified that having many sexual partners increases the risk of HPV. Furthermore, even though almost all the participants were aware of cervical cancer, about 60% did not know they were susceptible to this malignancy. Interestingly, nearly 40% believe they could be affected by cervical cancer in the future, this aspect presents an opportune avenue for translating awareness into proactive health behaviours and regular screenings as awareness of their susceptibility would make them proactive in terms of prevention.^20^ According to the Health belief model, perceived susceptibility to a dread disease like cancer is one of the factors that motivates a person to willingly engage in a health-seeking behaviour.^25^ Evidently, the public knowledge about cervical cancer and HPV vaccine has improved significantly in recent years partly because of the public awareness campaigns coinciding with the regulatory approval and public endorsements of HPV vaccines globally.^20^ However, the current reality is that cervical cancer will affect one in 26 women during their lifetime in South Africa, with many cases diagnosed late, especially among young women.^2,3,6^

### Study limitations

The current study has some limitations. Firstly, the use of a cross-sectional design hampers the ability to establish causal relationships and broad generalisations because of its snapshot nature. Longitudinal studies, which track knowledge levels over time, will be preferred in that instance. Additionally, the use of self-report questionnaires introduces biases related to participant motivation, recall accuracy and social desirability.

### Recommendations

Tailoring interventions to address the identified knowledge gaps among these university female students is essential for improving preventive measures against cervical cancer among this population. The following are hereby recommended:

- *Educational programmes*: The local health authority in collaboration with university management should develop and implement comprehensive educational programmes targeting female university students to increase their awareness of cervical cancer, its risk factors and the importance of early detection through Pap smear tests.
- *Tailored educational interventions*: Future health promotion programmes should ensure that specific attention is given to educating students about HPV, its role in cervical cancer and the availability of the HPV vaccine. In addition, there should be an emphasis on clear and accurate information regarding the HPV vaccine, its availability, affordability and its role in preventing cervical cancer. The programmes should address misconceptions and provide accurate information about the vaccine’s scope, timing, benefits and limitations.
- *Awareness campaigns*: The university should consider launching public health campaigns within the university settings to promote accurate knowledge about cervical cancer and HPV prevention, utilising various communication channels, including university health services, social media and educational workshops to disseminate accurate information and reach a broad audience.
- *Promote proactive health behaviour*: Efforts should be made to leverage the participants’ awareness of their susceptibility to cervical cancer in the future to encourage proactive health behaviours. It is essential to emphasise the importance of regular screenings and HPV vaccination as vital preventive measures for cervical cancer.

## Conclusion

In conclusion, it was evident that a substantial portion of the female university students exhibited a commendable level of knowledge and awareness concerning cervical cancer and the HPV vaccine. While some participants demonstrated a solid understanding of certain aspects of vaccination, there were notable misconceptions that need to be addressed. Accurate information dissemination, especially regarding the timing and scope of HPV vaccines, is essential for informed decision-making and maximising the impact of vaccination programmes in preventing cervical cancer. Ensuring a high level of awareness is crucial, as informed persons are more likely to participate actively in preventive healthcare measures. Secondly, the Pap smear test is regarded as the most important tool in the secondary prevention of cervical cancer. Capitalising on the existing high levels of cervical cancer awareness and the apparent inclination shown by these female students to undergo a Pap smear, by tailoring interventions within university settings, focusing on specific demographic groups with lower knowledge levels can play a pivotal role in promoting accurate knowledge and fostering a proactive approach towards HPV prevention. These efforts are vital for reducing the burden of cervical cancer, especially in regions with high morbidity and mortality rates.
